# A Case of Acute Granulomatous Conjunctivitis Caused by Cat-transmitted Sporothrix schenckii

**DOI:** 10.7759/cureus.3428

**Published:** 2018-10-08

**Authors:** Jiunn Loong L Ling, Koon Ling Koh, Evelyn Tai, Zakariah Sakinah, Yusof Nor Sharina, Adil Hussein

**Affiliations:** 1 Ophthalmology, School of Medical Sciences/Universiti Sains Malaysia, Kota Bharu, MYS; 2 Ophthalmology, Hospital Raja Perempuan Zainab, Kota Bharu, MYS

**Keywords:** sporothrix schenckii, conjunctiva, conjunctivitis, itraconazole, sporotrichosis

## Abstract

In human, sporotrichosis infection commonly manifests as skin lesions through direct inoculation. It is rarely associated with ocular manifestation via a zoonotic transmission. We describe a young lady who presented with acute left eye granulomatous conjunctivitis who had a history of exposure to her sick cat diagnosed with sporotrichosis infection. Sporothrix schenckii was isolated from the culture of the excised conjunctival tissue. The patient recovered fully after six months of oral anti-fungal treatment. Clinicians should be aware of this new zoonotic infection transmitted by infected felines as it is reversible with timely diagnosis and initiation of anti-fungal therapy.

## Introduction

The dimorphic fungus Sporothrix schenckii is the causative organism for sporotrichosis. It is found in soil, plants, and organic matter of the tropical or subtropical region. Infections in human are usually restricted to the skin, subcutaneous cellular tissue, and adjacent lymphatic vessels [1]. Rarely, the organism may also disseminate to other tissues in the body. Approximately 5%-10% of the cases are characterized by more severe diseases including mucosal, disseminated, and extracutaneous (pulmonary, osteoarticular, etc.) forms [2]. We report a rare case of sporotrichosis affecting the palpebral conjunctiva in an owner of a cat with atraumatic exposure to asporotrix-infected cat.

## Case presentation

An 18-year-old lady with no medical comorbidities presented to the eye clinic with complaints of left eye redness and pain for three days associated with several small nodular lesions on her left inferior palpebral conjunctiva. However, there was no blurring of vision, eye discharge or itchiness. She is a cat lover and had been caring for her sick cat. Despite veterinary care, her cat subsequently died because of sporotrichosis infection. However, she denied any history of a cat scratch, trauma, or contact with organic matter. She also denied any fever, skin infection, or respiratory symptoms.

**Figure 1:**
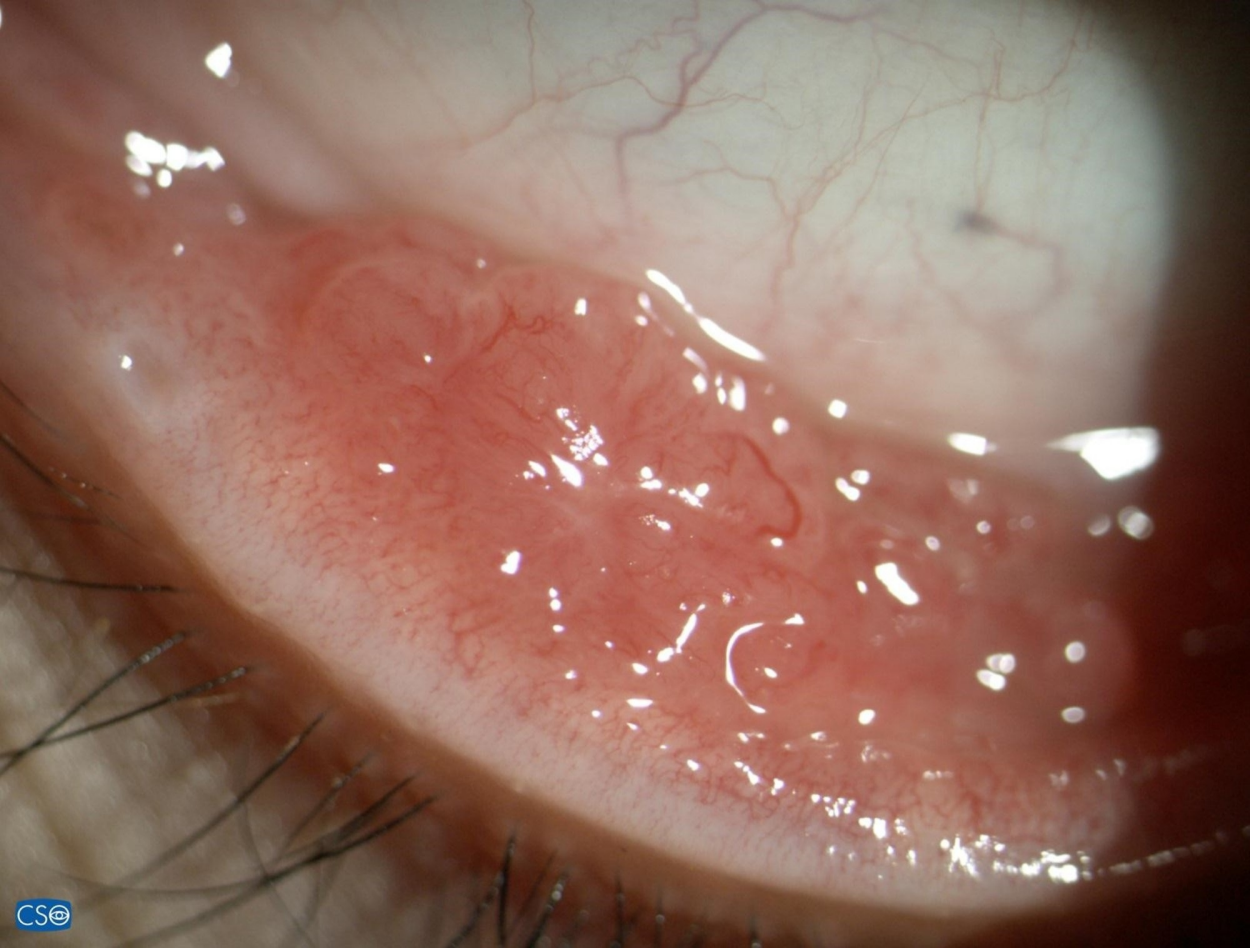
Granulomatous lesions on lower palpebral conjunctiva

**Figure 2:**
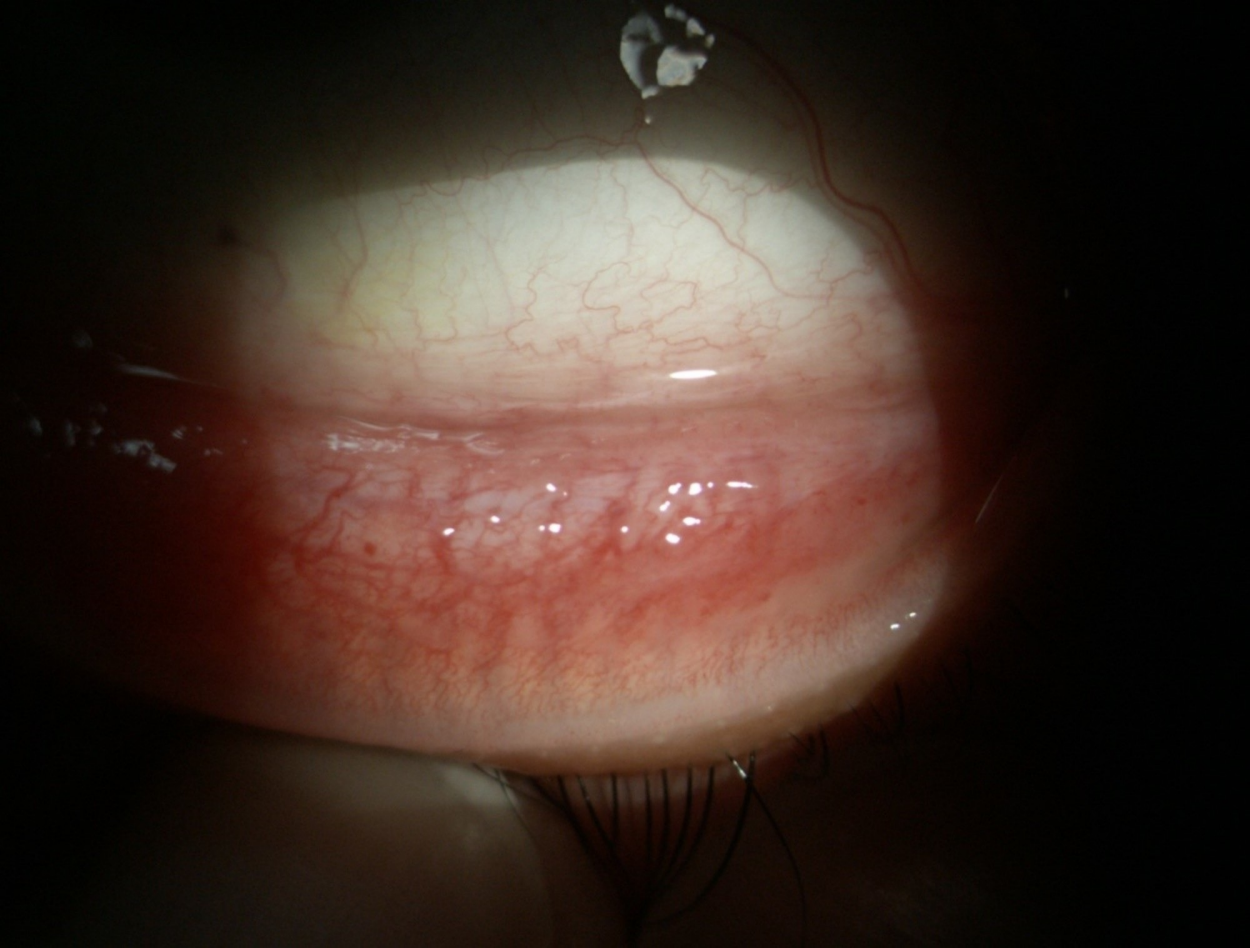
Resolved granulomatous lesion on inferior palpebral conjunctiva

On examination, the patient’s visual acuity was found to be 6/9 in both eyes. Her left eye showed conjunctival hyperemia with generalized granulomatous lesions over the superior and inferior palpebral conjunctiva (Figure 1). The granulomatous conjunctival lesions were covered with thin whitish discharge. The cornea was normal, as was the anterior chamber. Posterior segment was likewise unremarkable. Systemic examination revealed a swollen and painful left cervical lymph node measuring about 1 x 2 cm. The patient was afebrile. There was no evidence of cutaneous fungal infection. An excisional biopsy of the left eye inferior conjunctival fornix lesion was performed. The patient was started on guttate fluconazole q1h and guttate ciprofloxacin q2h on the left eye while waiting for the tissue histopathology and culture results. Tissue specimen identified numerous granulomas with few fungal yeasts engulfed by histiocytes. Culture of the tissue isolated S. schenckii. The topical fluconazole and ciprofloxacin were stopped and the patient was treated with oral itraconazole 200 mg twice daily for six months. Her condition improved gradually and the conjunctival lesions completely resolved after five months of treatment (Figure 2).

## Discussion

The aetiological agent for sporotrichosis, S. schenckii commonly penetrates into human skin by traumatic inoculation of organic matter contaminated with the fungus [ 1]. The prevalence is thus higher among gardeners, farmers, woodcutters and miners, where it is frequently referred to as ‘rose-bush mycosis’ or ‘rose-gardeners' disease [3]. A small number of zoonotic transmissions have been reported, in which armadillos and cats are the animals most frequently involved [4]. 

Although sporotrichosis occurring in felines has been considered a rare disease, cats are emerging as an alternative route of transmission of S. schenckii to humans  [5]. 

Duration 1998 to 2001, there was an outbreak of culture-proven sporotrichosis associated with transmission by cats in 178 patients in Rio de Janeiro, Brazil. Of these cases, 80.9% presented with isolated cutaneous or lympho-cutaneous form of the disease, 2.2% had conjunctival involvement, and 0.6% presented with nasal fossa involvement [4]. According to Barros et al., the most common clinical form of sporotrichosis is lympho-cutaneous disease, which accounts for approximately 75% of cases, followed by localized cutaneous forms (20%) [1].

Ocular mucosal involvement such as conjunctivitis or episcleritis are rare and are almost always induced by trauma, while intraocular involvement in sporotrichosis occurs mainly from hematogenous dissemination [6]. Primary conjunctival sporotrichosis, in the absence of direct traumatic inoculation, is described in only three cases in the literature [6-7]. Reported posterior segment or adnexal manifestations of S. schenckii include endophthalmitis, anterior uveitis, retinal granuloma, and acute dacryocystitis [8-10]. Immunocompromised patients may have a more extensive and severe clinical presentation. Simultaneous involvement of the conjunctiva and the regional lymph nodes is known as Parinaud syndrome, which was seen in our patient [11].

Oral itraconazole, a fungistatic drug, is considered the treatment of choice in cutaneous and extracutaneous sporotrichosis due to its effectiveness, safety and is classified as having an AII scientific evidence level [12]. It may be used in healthy patients with limited lesions, as well as in immunosuppressed patients in the systemic form. Alternative therapy includes terbinafine or amphotericin B in severe, life-threatening sepsis. For cutaneous or lympho-cutaneous sporotrichosis, treatment must be continued until the clinical cure is reached, which usually occurs within two to three months. However, ocular and other systemic forms require longer treatment, ranging from six to twelve months. Our patient achieved clinical cure after five months and the treatment was discontinued after she had completed a total of six months on oral itraconazole.

## Conclusions

Sporotrichosis, a predominantly cutaneous fungal disease, may manifest primarily with granulomatous conjunctivitis, without concurrent cutaneous lesions. Clinicians should be aware of this new zoonosis transmitted by infected felines, and administer timely treatment to prevent complications such as symblepharon or conjunctival fibrosis.
